# Human Endothelial-Like Differentiated Precursor Cells Maintain Their Endothelial Characteristics When Cocultured with Mesenchymal Stem Cell and Seeded onto Human Cancellous Bone

**DOI:** 10.1155/2013/364591

**Published:** 2013-02-17

**Authors:** Dirk Henrich, Kerstin Wilhelm, Joerg Warzecha, Johannes Frank, John Barker, Ingo Marzi, Caroline Seebach

**Affiliations:** Department of Trauma, Hand and Reconstructive Surgery, Johann Wolfgang Goethe University, Theodor-Stern-Kai 7, 60590 Frankfurt, Germany

## Abstract

*Introduction*. Cancellous bone is frequently used for filling bone defects in a clinical setting. It provides favourable conditions for regenerative cells such as MSC and early EPC. The combination of MSC and EPC results in superior bone healing in experimental bone healing models. *Materials and Methods*. We investigated the influence of osteogenic culture conditions on the endothelial properties of early EPC and the osteogenic properties of MSC when cocultured on cancellous bone. Additionally, cell adhesion, metabolic activity, and differentiation were assessed 2, 6, and 10 days after seeding. *Results*. The number of adhering EPC and MSC decreased over time; however the cells remained metabolically active over the 10-day measurement period. In spite of a decline of lineage specific markers, cells maintained their differentiation to a reduced level. Osteogenic stimulation of EPC caused a decline but not abolishment of endothelial characteristics and did not induce osteogenic gene expression. Osteogenic stimulation of MSC significantly increased their metabolic activity whereas collagen-1*α* and alkaline phosphatase gene expressions declined. When cocultured with EPC, MSC's collagen-1*α* gene expression increased significantly. *Conclusion*. EPC and MSC can be cocultured *in vitro* on cancellous bone under osteogenic conditions, and coculturing EPC with MSC stabilizes the latter's collagen-1*α* gene expression.

## 1. Introduction 

Autologous cell transplantation is a promising treatment option for large bone defects as it eliminates problems such as limited autologous bone availability, allogenic bone immunogenicity, and donor site morbidity and could be used for stabilizing loose alloplastic implant [1, 2]. Marrow-derived stromal cells (MSCs) alone and in combination with endothelial progenitor cells (EPCs) have been shown to promote bone healing in a variety of different settings [3–5]. 

The bone formation was shown to be improved when MSCs were preconditioned with osteogenic substances such as ascorbic acid and dexamethasone [6]. Another study demonstrated an enhanced callus formation after transplantation of osteogenic predifferentiated MSC during distraction osteogenesis in 3 patients [7]. In an animal study dexamethasone-pretreated MSC seeded on a collagen sponge result in a significant higher mineralization of the collagenous matrix. The mineralization could be clearly ascertained to the transplanted cells [8].

Improved bone healing has also been described in the presence of EPC. This effect has been attributed to EPC stimulation of early vascularization, a prerequisite for *in vivo* bone regeneration [9]. At least two major types of endothelial cell lines can be obtained by *in vitro* culture of mononuclear cells: first, the so-called “endothelial-like cells” or “early EPC” and, second, the so-called “outgrowth EPC” or “late EPC”. Early EPCs are derived presumably from monocytic/dendritic precursors, and some authors therefore designate them as endothelial-like differentiated PBMC [10, 11]. Those cells can be generated in a sufficient amount within 3 to 5 days from a reasonable volume of blood [12]. Early EPCs are potent producers of vascular endothelial growth factor (VEGF) [13]. 

When transplanting MSC and/or EPC into bone defects, a scaffold is needed. Synthetic or processed bone-graft substitutes should be osteoinductive, enabling the osteogenic differentiation of cells, should provide appropriate mechanical stability, should permit the ingrowth of cells and vessels [14] thereby improving bone regeneration [15], and should be resorbable. Various porous ceramics are currently available [16]. Hydroxyapatite (HA) sintered ceramics are widely used due to their osteoconductivity but their bioresorbability are comparatively low. In contrast, tricalcium phosphate (TCP) ceramics were porous, resorbable, and biocompatible materials. They do not provoke an inflammatory response and permit the ingrowth of cells and vessels [17] during bone regeneration. Furthermore, TCP can be completely substituted for the bone tissue after stimulation of bone formation. Also the surface chemistry of the scaffold influences the behaviour, through either the influence of its charge density or atomic array on adherent or passing cell populations [18]. Moreover, the surface charge of the biomaterials influences the binding of matrix proteins or growth factors, which might also influence the cell behaviour locally [19]. 

Bone allografts consist of a collagen fibre network with attached hydroxyapatite crystals providing elasticity and perhaps osteoconductive properties to these scaffolds [20]. Those materials do not evoke any appreciable foreign-body immunogenic reaction. Although antigenic structures were destroyed during processing components of the extracellular matrix, various growth factors such as bone morphogenetic protein-2 (BMP-2) remain functionally active [21, 22]. 

Bone graft substitutes are well described physically and chemically [23] as an osteoconductive scaffold, but even more of interest are biological properties like cell adhesion and function of processed and synthetic biomaterials as delivery system for bone tissue engineering in critical size defects. However, depending on the method used to process the bone allografts the active (osteoinductive) and passive (osteoconductive) biologic interaction between the scaffold and the transplanted cells may vary and greatly influence the proliferation of the latter [24]. This was proved in a comparative study in which a markedly increased survival and metabolic activity of MSC on human cancellous bone (*Tutoplast*) in comparison to five different synthetic materials were observed [20].

Recently it was shown that EPC and MSC can be successfully grafted onto a *β*-TCP-matrix [25]; however, little data exists regarding the survival, proliferation, and differentiation of these cells when seeded on human cancellous bone (*Tutoplast*). 

A beneficial effect on bone healing mediated by osteogenic preconditioned MSC has been repeatedly described [6–8]. But the effect of the osteogenic conditions on early EPC has not been addressed. Moreover, the influence of osteogenic culture conditions on the number, metabolic activity, and differentiation of early EPC alone or in coculture with MSC has not been addressed yet. 

In the present study we investigated the influence of osteogenic culture conditions on the endothelial properties of human early EPC seeded *alone* on human cancellous bone chips and *together* with human MSC.

## 2. Materials and Methods

### 2.1. Isolation, Cultivation, and Characterization of MSC

Bone marrow cells were obtained from iliac crest aspirates of volunteer trauma patients (*n* = 5) undergoing pelvic surgery [26]. This was performed in accordance with and with the approval of our hospital's ethics committee. All patients signed informed consent. Briefly, MSCs were isolated from fresh bone marrow aspirate using Ficoll density gradient centrifugation (30 min, 1100 g, *d* = 1,077 g/mL, Biochrom, Berlin, Germany). Cells in the interphase were collected, washed twice with PBS containing 2% fetal bovine serum (FBS) (10 min, 900 g), resuspended in 3 mL *MesenCult* + Supplements (Cell-Systems, St. Katharinen, Germany), and were counted using a *Neubauer* chamber. 4 × 10^6^ cells were seeded in a 25 cm^2^ culture flask and then expanded over three to five passages prior to being used in the present experiments. Cells were detached by 10 min incubation with Accutase, then washed (10 min, 300 g), re-suspended in *MesenCult* + Supplements, and divided in 2 parts. One part was adjusted to a density of 2.5 × 10^5^ cells in 100 *μ*L and used for the present experiments, and the other was used for confirmation of MSC, surface characteristics using flow cytometry (FACSCalibur, BD-Biosciences, Heidelberg, Germany). MSCs were negative for CD45 and CD34 and express CD73, CD90, and CD105 as described previously; all antibodies were purchased from BD-Biosciences [26]. 

### 2.2. Isolation, Identification, and Characterization of Early EPC

EPCs were isolated according to procedures described elsewhere [25, 27]. Briefly, PBMCs were isolated from buffy coat (*n* = 5) by density gradient centrifugation (20 min, 600 g) with Ficoll (1.077 g/mL, Biochrom, Berlin, Germany). PBMCs were washed twice with cold PBS without Ca^2+^ and Mg^2+^ (PBS^w/o^, 10 min, 350 g), and 4 ∗ 10^6^ cells were cultivated on a fibronectin-coated (10 *μ*g/mL, Sigma, Deisenhofen, Germany) 24-well culture dish in 1 mL of endothelial basal medium (EBM2, Cambrex, Verviers, Belgium) supplemented with EGM2 SingleQuots at 37°C, 5% CO_2_. After 48 hrs, non- and weakly adherent cells were removed, the medium was exchanged, and the cells were cultivated for an additional 72 hrs. A parallel preparation was performed to evaluate the percentage of endothelial cell-like differentiated cells. Cells were incubated for 1 h with 2.4 *μ*g/mL 1,1′-dioctadecyl-3,3,3′,3′-tetramethylindo-carbocyanine-labeled acetylated low-density lipoprotein (DiLDL, CellSystems, St. Katharinen, Germany) in EBM supplemented with 20% FCS. Cells were fixed with 2% paraformaldehyde for 10 min, and after washing with PBS^+/+^ FITC-labelled Ulex europaeus agglutinin-1 [10 *μ*g/mL] (lectin, Sigma, Deisenhofen, Germany) was incubated for 1 h. Cells presenting double-positive fluorescence were considered to be EPC. Only preparations with a percentage of endothelial like differentiated cells of greater than 80% were used. For the determination of the expression of the endothelial markers CD31 and von Willebrandt factor (vWF) a volume of 100 *μ*L DiLDL prestained cells was incubated for 20 min with either monoclonal antibody against vWF (Dako, Hamburg, Germany) followed by 15 min incubation with a FITC conjugated secondary antibody (Dako) or with a FITC-conjugated monoclonal antibody against CD31 (Chemikon, Hofheim, Germany). Antibodies of identical isotypes served as control. After two steps of washing, the cells were subjected to flow cytometry using a *FACSCalibur* (BD Biosciences). 

Cells were detached by incubation (10 min) with Accutase (PAA Laboratories, Linz, Austria), washed once with MesenCult + Supplements (Cell-Systems, St. Katharinen, Germany) and then adjusted to a density of 2.5 × 10^5^ cells in 100 *μ*L.

### 2.3. Human-Processed Cancellous Bone (*Tutoplast*)

A processed human cancellous bone allograft material (*Tutoplast*, Tutogen Medical) was used. The solvent preserved, dried, and gamma-irradiated material has a particle size of 0.5–2 mm with a pore size ranging from 100 to 500 *μ*m. The material has a high mechanical stability and a rapid biodegradability (information provided by the manufacturer).

### 2.4. Seeding Cells on Scaffold

*Tutoplast* granules were placed as a dense single layer in a 24-well plate (Nunc, Wiesbaden, Germany) using sterile forceps and prewetted with 200 *μ*L PBS with Ca^2+^ and Mg^2+^ (PBS^+/+^). 5 × 10^5^ MSCs or EPCs in a volume of 200 *μ*L were dripped onto the *Tutoplast* layer and were incubated for 10 min at 37°C. When MSC together with EPCs were tested, 2.5 × 10^5^ cells of each were mixed immediately prior to seeding. 

After incubation, a medium containing the nonadhering cells was removed and dripped once again over the *Tutoplast *layer, followed by incubation as indicated above. This procedure was repeated three times. The granules were then gently transferred to another well containing 500 *μ*L of a mixture consisting of 2 parts *Mesencult* + supplements and 1 part *EBM2*+*EGM2 Singlequots*. Below this mixture is referred to as the “medium” and is described in [25]. All experiments were performed using the said medium. The remaining cells in the supernatant and the bottom of the initial seeding well were isolated, counted, and the percentage of adhering cells was calculated ((initial cell number − remaining cell number)/initial cell number) ∗ 100.

### 2.5. Identification of MSC and Early EPC on *Tutoplast*

MSC and EPC, alone and combined, were seeded on *Tutoplast* as described above. Approximately 4-5 granules were transferred to a single well of a 96-well plate using sterile forceps and incubated in 100 *μ*L medium for a period of 2, 6 or 10 days in a CO_2_ incubator at 37°C. Parallel preparations included the same as above with the addition of substances that promote osteogenic differentiation: Dexamethasone [final concentration 1.0 × 10^−7^ M], *β*-glycerolphosphate [final concentration 1.0 × 10^−2^ M], and ascorbic acid [final concentration 5 × 10^−5^ M] [6]. All substances were obtained from Sigma (Deisenhofen, Germany). In order to detect MSC and EPC, MSC 1 *μ*L of DiLDL (CellSystem, St. Katharinen, Germany) or 1 *μ*L DAPI [final concentration 1 *μ*g/mL] was added to the corresponding wells followed by further incubation for 1 h at 37°C. After three washes with PBS^−/−^, the granules were transferred to a new well in order to prevent false-positive results caused by cells adhering to the bottom of the cultivation well. Finally, randomly chosen *Tutoplast* granules were viewed at 100x magnification using fluorescence microscopy (Zeiss, Axio Observer, Gottingen, Germany), and the DiLDL-stained EPC and DAPI-stained MSC were counted using *cell explorer 2001* (BioScieTec, Frankfurt, Germany) software.

### 2.6. Identification of EPC and MSC by Scanning Electron Microscopy (SEM)

*Tutoplast* granules were seeded with either MSC, EPC, or both. After 2 days the cell-*Tutoplast* constructs were fixed in 2% glutaraldehyde in PBS (Merck, Darmstadt, Germany) for 30 min and dehydrated by incubation for 15 min in PBS with increasing concentrations of ethanol (25%, 50%, 75%, 96%, and 100%). Subsequently the samples were incubated overnight in 1,1,1,3,3,3-hexamethyldisilazane (Merck, Darmstadt, Germany) and slowly air-dried. The granules were fixed with carbon glue to an aluminium carrier and sputtered trice for each 1 min with gold (*Agar Sputter Coater*, Agar Scientific Ltd., UK) and subjected to scanning electron microscopy. A *Hitachi FE-SEM S4500* was used (Hitachi, Düsseldorf, Germany). A voltage of 5 kV was applied. The images were digitally recorded using the *Digital Image Processing System *2.6 (Point Electronic, Halle, Germany).

### 2.7. Cell-*Tutoplast* Adherence (MTT Assay)

For determination of the metabolic activity as a correlate of the number of cells adhering to the *Tutoplast* scaffold a *Cell proliferation Kit I *(*MTT*, Roche Diagnostics, Mannheim, Germany) was used following the manufacturer's instructions. In brief, the MTT assay is based on the cleavage of the yellow tetrazolium salt MTT (3-[4,5-Dimethylthiazol-2-yl]-2,5-diphenyltetrazolium bromide) to purple formazan crystals by metabolically active cells. MSC obtained from 5 donors and EPC derived from 5 buffy coats were tested. The following combinations were performed: MSC alone, EPC alone, and MSC and EPC together. All experiments were performed in duplicate.

Cells were seeded on *Tutoplast*, as described above, and 3 to 5 granules, depending on the granule's size, were transferred to new wells in a 96-well plate (Nunc, Roskilde, Denmark) and cultivated for 2, 6, and 10 days. Before adding the MTT reagent, granules were transferred to an empty well, in order to prevent false positive results caused by cells adhering to the bottom of the well. 90 *μ*L of medium and 10 *μ*L of MTT labelling reagent were added to each well and cells were incubated for an additional 4 hrs. Next, the cells were incubated overnight with a solubilization solution. The supernatant was collected and transferred to another 96-well plate. The absorbance at 570 nm was then measured with an ELISA reader (Ceres UV900c, Bio-Tek Instruments, Windoski, VT, USA). As controls, increasing numbers (1000, 2500, 5000, and 10.000) of MSC and EPC were seeded directly in 96 well plates and assessed separately. 

### 2.8. Expression of Osteogenic and Endothelial Marker Genes by Real Time RT-PCR

In brief, total RNA was isolated using the *RNeasy* system (Qiagen, Hilden, Germany) following the manufacturer's instructions with the following exception. Approximately 50 *μ*L *Tutoplast*-granules that had been sown with progenitor cells was incubated in *RLT* buffer for 3 min, the mixture was gently vortexed, and the supernatant was subjected to the RNA isolation procedure. The quality and quantity of RNA was determined using the *NanoDrop* ND-1000 device (Nanodrop technologies, Wilmington, Delaware, USA). Contaminating genomic DNA was removed by digestion with the *RNase-free DNase Kit* following the manufacturer's protocol (Qiagen). 

Each 250 ng of RNA was reversely transcribed using an *Affinity script QPCR-cDNA synthesis kit* (Stratagene, La Jolla, CA, USA) following the manufacturer's instructions. 

Real time RT-PCR was performed on a *Stratagene MX3005P QPCR system* (Stratagene, La Jolla, CA, USA). PCR was performed using the primer assays for human collagen-1 (COL1A, NM_000088, catalog number PPH01299F), alkaline phosphatase (ALPL, NM_000478, catalog number PAHS-026), core binding factor-1 (cbfa-1 also known as Runt-related transcription factor 2, RUNX2, NM_004348.3, catalog number PPH01897B), osteocalcin (BGLAP, bone gamma-carboxyglutamate (gla) protein, NM_199173.3, catalog number PPH01898A), vascular endothelial growth factor (VEGF, NM_003376.4, catalog number PPH00251B), and von Willebrand factor (vWF, NM_000552.3, catalog number PPH02567E); all purchased from Biomol (SuperArray, Frederick, MD, USA). As reference gene the expression of glyceraldehyde-3-phosphate dehydrogenase (GAPDH, NM_002046.3, catalogue number PPH00150E) was measured. 

A melting curve analysis was applied to ensure the specificity of the PCR reaction. Relative quantification of the mRNA levels of the target genes was determined using the comparative CT (threshold cycle values) method (2^−ΔCT^ method). The results are presented as fold change to GAPDH gene expression.

### 2.9. Statistics

Results are presented as mean values and standard error of mean (SEM). Kruskal-Wallis test with Dunn's post hoc test for multiplicity was used for comparisons between the groups and for the analysis of changes during the follow-up period (day 2 versus day 6 and day 10). A *P* value < 0.05 indicates statistical significance.

## 3. Results

### 3.1. MSC and EPC Characterization

**Figure 1:**
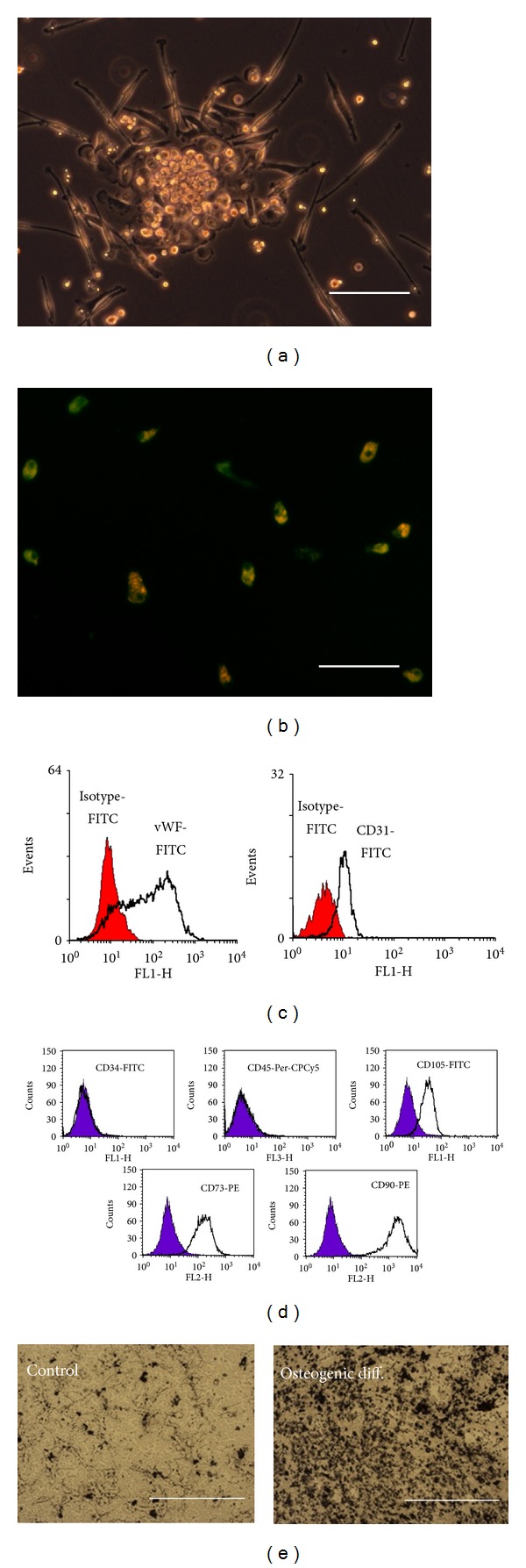
Characterization of EPC (a–c) and MSC (d, e). Representative results were shown. EPC demonstrated a spindle-shaped appearance (a). The ability of EPC for DiLDL uptake (red color) and the binding of UEA-lectin-FITC (green color) are shown in (b). The image consists of a superimposition of the FITC-fluorescence channel and the Rhodamine-fluorescence channel. Cells carrying both characteristics appear in orange. The protein expression of vWF (c) and CD31 (PECAM, (c)) was assessed by flow cytometry (filled histograms demonstrate the isotype control; black line indicates the antibody staining). The MSCs were characterized by flow cytometry (d); the cells were negative for CD34 and CD45 and express CD73, CD90 (Thy-1), and CD105 (Endoglin, filled histogramms demonstrate the isotype control; black line indicates the antibody staining). In (e) the ability of MSC for osteogenic differentiation was assessed by means of *von Kossa staining*. The left image demonstrated the calcium deposition (black spots) under control conditions; the right image depicted the calcium deposition under osteogenic culture conditions. Scale bars represent 50 *μ*m (a), 100 *μ*m (b), and 500 *μ*m (e).

EPC showed typical early EPC characteristics such as spindle-shaped appearance, DiLDL uptake, binding of UEA-1-lectin, and the expression of vWF and CD31 (Figures 1(a)–1(d)). MSC from all donors had a fusiform and a spindle-shaped appearance and demonstrated a typical pattern of surface markers (CD34−, CD45−, CD71+, CD90+, CD105+). Moreover, osteogenic differentiation was inducible as assessed by *van Kossa* staining (Figures 1(d) and 1(e)).

### 3.2. MSC and EPC Adherence to *Tutoplast*

The overall percentage of initially adhering cells ranged from 90% to 95% and did not differ significantly between MSC and EPC. 

**Figure 2:**
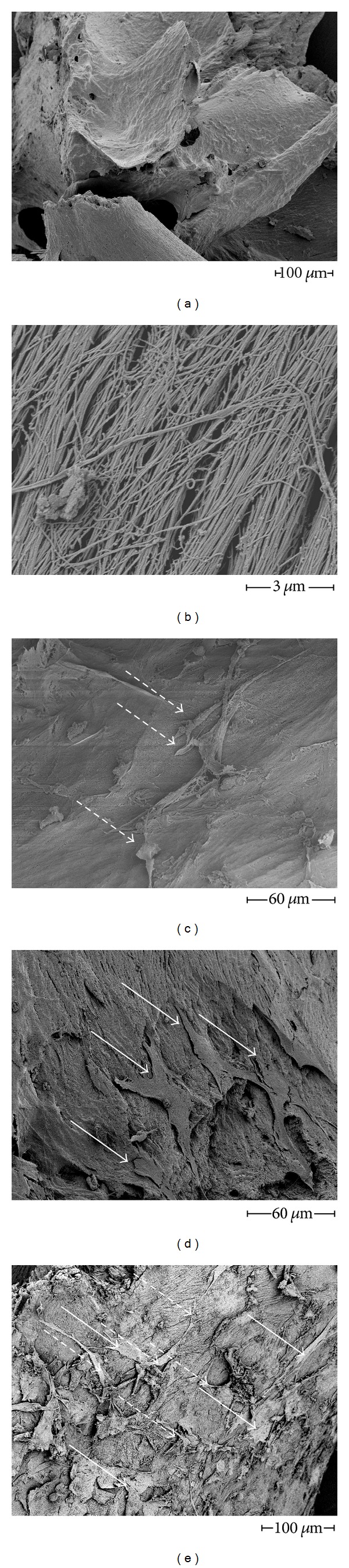
Surface characteristics and direct proof of EPC and MSC on *Tutoplast* by SEM. (a) shows a *Tutoplast* granule without cells, (b) in a higher magnification to demonstrate the fibrillar structure. (c), (d), and (e) show *Tutoplast* sown with EPC (c), with MSC (d), and with both cell types in combination (e). Early EPC appeared rather spindle shaped and elongated whereas the MSC demonstrated a more flattened and out-spread phenotype. Scanning electron microscopy was performed as described in the Materials and Methods Section. Bold type arrows indicate MSC, and spotted arrows indicate EPC. The scale bar indicates 3 *μ*m (b), 60 *μ*m (c, d), and 100 *μ*m (a, e), respectively.

Scanning electron microscopy images of *Tutoplast* 2 days after cell seeding revealed a slightly roughened surface lacking sharp edges (Figure 2(a)). Higher magnification revealed a surface composed of a dense layer of fibrils (Figure 2(b)). Both MSC and EPC appeared firmly attached to the *Tutoplast* surface. While MSC had a bigger size than EPC and showed a flattened and spread-out appearance (Figure 2(d)), EPC appeared elongated and spindle shaped (Figure 2(c)). In the samples containing both MSC and EPC their district shapes made it possible to differentiate between the 2 cell types on the *Tutoplast* surface (Figure 2(e)).

### 3.3. Number of Adhering MSC and EPC on *Tutoplast* Surface

**Figure 3:**
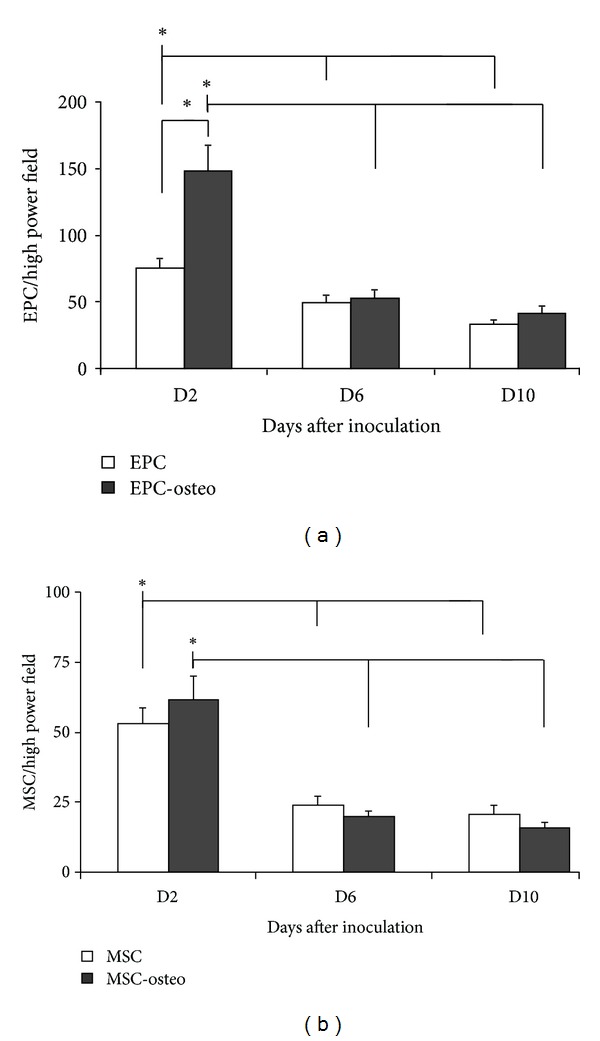
Adhesion of EPC and MSC on *Tutoplast*. The number of adhering EPC (a) and MSC (b) cultivated on *Tutoplast* declines significantly over the time independently of the presence of osteogenic substances. *: *P* < 0.05.

In the samples containing both MSC and EPC, the number of EPC declined significantly (*P* < 0.05) from day 2 to day 6 and then remained stable until day 10. This was also observed in samples in which EPCs were incubated in the presence of osteogenic supplements. However, on day 2 after seeding, the number of adhering EPCs was significantly higher in cultures supplemented with osteogenic factors (Figure 3(a)). 

The number of adhering MSCs also decreased significantly (*P* < 0.05) from day 2 to days 6 and 10, independent of the presence of osteogenic factors (Figure 3(b)).

### 3.4. MSC and EPC: Metabolic Activity

**Figure 4:**
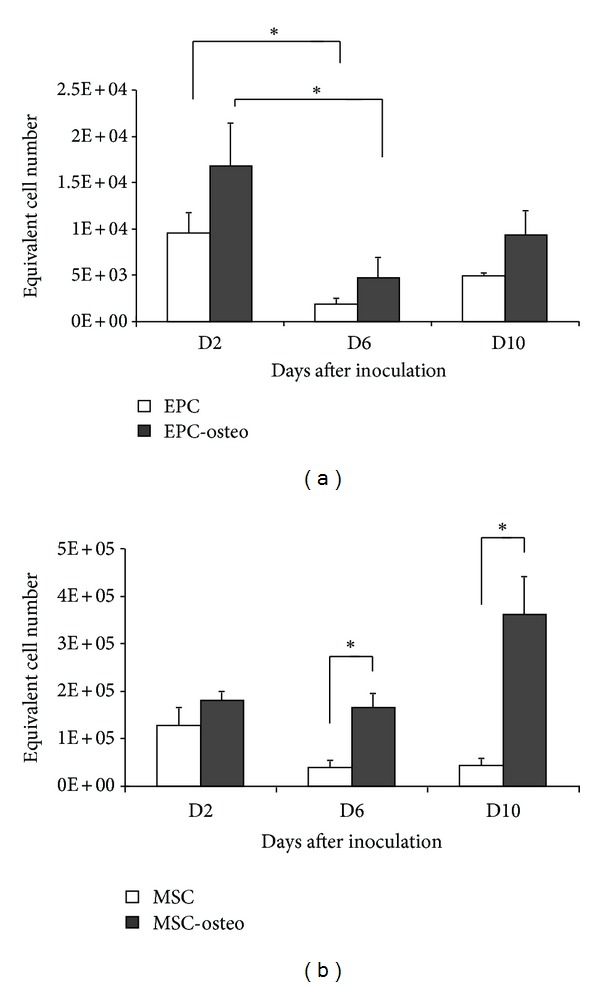
MTT assay, metabolic activity of EPC (a) and MSC (b) cultivated on *Tutoplast*. Presence of osteogenic substances lead to a significant increase in MTT conversion in MSC. The metabolic activity was assessed using the MTT assay as described in Section 2.  *: *P* < 0.05.

The MTT assay demonstrated MSC and EPC metabolic activity over the entire observation period. The number of adhering cells paralleled the metabolic activity of EPC, dropping significantly (*P* < 0.05, Figure 4(a)) from day 2 to day 6. In the presence of osteogenic supplements the metabolic activity of EPC slightly increased in comparison to controls; however, this increase was not statistically significant (Figure 4(a)).

The metabolic activity of MSC decreased slightly with time, depicting a similar course as described for the number of adhering MSCs. This decrease was not statistically significant (Figure 4(b)). In contrast to EPC, when osteogenic substances were added to MSC, metabolic activity was significantly increased compared to the corresponding controls on day 6 and day 10 (Figure 4(b)). 

### 3.5. Endothelial Differentiation (DiLDL Uptake)

**Figure 5:**
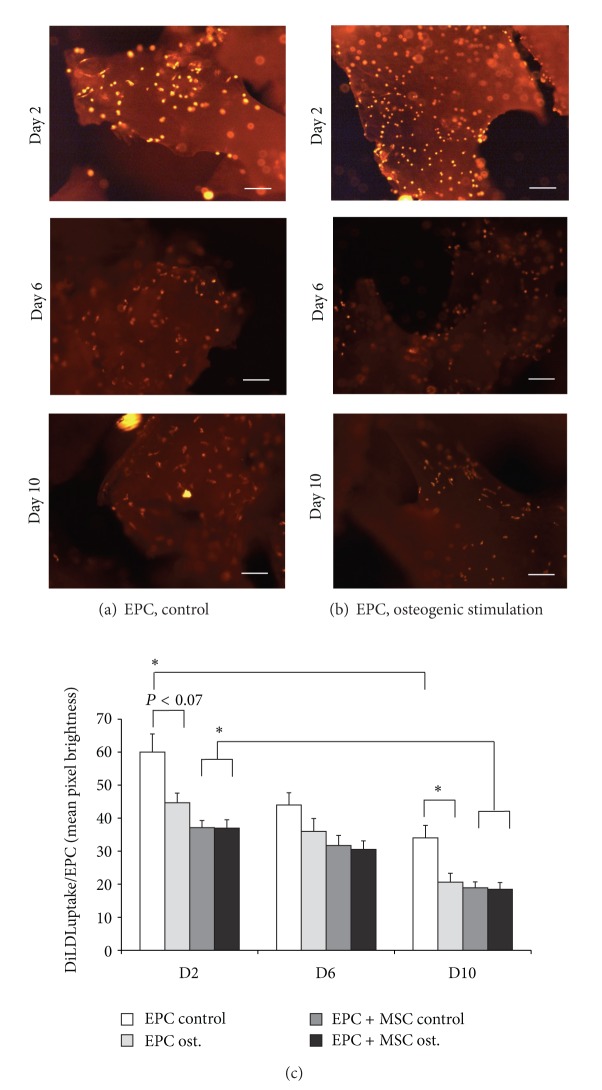
EPC on *Tutoplast*, decline of DiLDL uptake over the observation period independently of the presence of osteogenic stimulation. Representative images of day 2, day 6, and day 10 under control conditions (a) and osteogenic conditions (b), and data evaluations (c) were shown. Parallel preparations were used. The DiLDL staining was performed on the day of measurement. Thus, a reduced DiLDL fluorescence is not due to a fading effect but due to a reduced uptake of DiLDL. The exposure time was 0.25 s. Scale bar indicates 100 *μ*m.

 The uptake of acetylated low-density lipoprotein is an active process that characterizes endothelial cells. Although a significant (*P* < 0.05) decline of the DiLDL uptake was noted over the time, a significant ability of EPC to uptake DiLDL was conserved over the entire 10-day observation period (Figure 5(a)). Using an exposure time of 0.25 s the mean pixel brightness decreased from 60.0 ± 5.5 at day 2 to 34 ± 3.8 at day 10 (*P* < 0.05 versus day 2, Figures 5(a) and 5(c)). 

Under osteogenic conditions the DiLDL uptake was impaired on day 2 (trend: *P* < 0.07) and day 10 (*P* < 0.05) in comparison to the corresponding controls (Figures 5(b) and 5(c)). 

In samples where MSC and EPC were cocultured the mean pixel brightness between days 2 and 10 declined significantly (*P* < 0.05, Figure 5(c)). However, the mean pixel brightness on day 2 was lower in comparison to EPC cultured alone on *Tutoplast* (37.1 ± 2.2 versus 60.0 ± 5.5, *P* < 0.05, Figure 5(c)).

In co-culture and under osteogenic conditions a comparable DiLDL fluorescence was observed, that declined significantly over 10 days (*P* < 0.05, Figure 5(c), day 2: 37.0 ± 2.5, day 6: 30.5 ± 2.6, and day 10: 18.5 ± 2.0). 

### 3.6. MSC and EPC Differentiation (Gene Expression Analysis)

**Figure 6:**
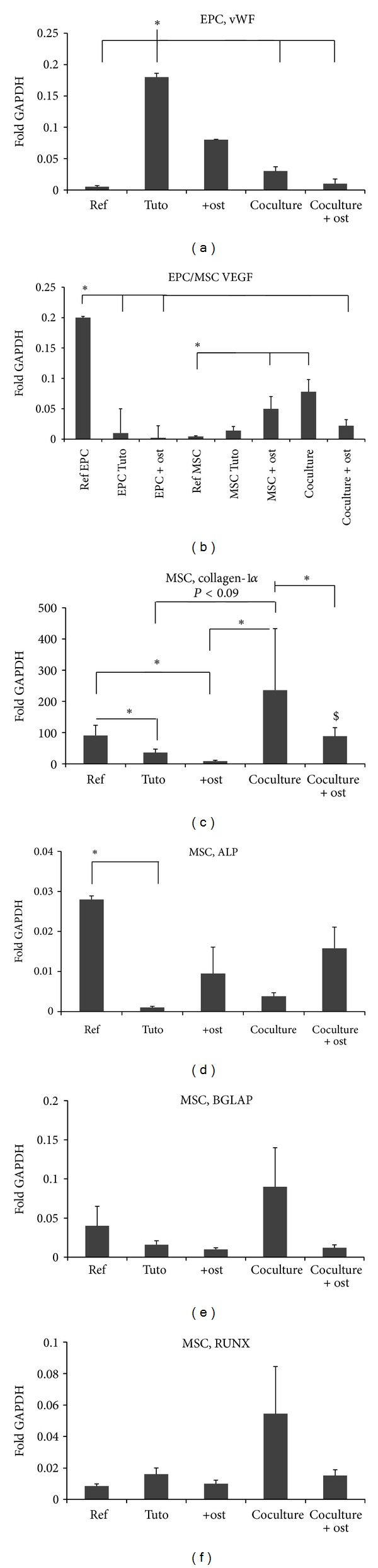
Expression of endothelial and osteogenic marker genes after 10 days on *Tutoplast*. Results of the real-time RT-PCR for vWF in EPC (a), VEGF in EPC and MSC (b), collagen-1*α* (c), alkaline phosphatase (ALP) (d), osteocalcin (BGLAP) (d), and the transcription factor cbfa-1 (RUNX) (e) in MSC are shown. Messenger RNA was isolated from either MSC, EPC, or MSC and EPC cultured on *Tutoplast* for 10 days. Reference RNA derived from EPC and MSC cultured under normal conditions was analyzed as well. Five MSC lines, respectively, five EPC lines were tested. Experiments were performed in duplicate. Gene expression was determined by the comparative ΔCt method as described in Section 2. Data are expressed as mean ± SEM of fold change to GAPDH gene expression. *: *P* < 0.05; $: *P* < 0.05 coculture (osteogenic conditions) versus MSC (osteogenic conditions).

In order to assess MSC and EPC differentiation expression of osteogenic genes (Collagen-1alpha, RUNX-2, BGLAP, ALP) and endothelial marker genes (vWF, VEGF) were measured on day 10. vWF gene expression significantly (*P* < 0.05) increased when EPCs were cultured on *Tutoplast* versus the culture on plastic (reference EPC) (Figure 6(a)). Osteogenic stimulation of EPC led to a decrease in vWF gene expression (*P* = 0.09, Figure 6(a)). 

In contrast, VEGF gene expression was significantly (*P* < 0.05) higher in EPC cultured on plastic as well as on *Tutoplast* under osteogenic conditions versus EPC cultured with *Tutoplast* alone (Figure 6(b)).

Collagen-1*α* and ALP gene expression was limited to MSC whereas a slight gene expression of RUNX and BGLAP was also observed in EPC independent from the culture conditions. Collagen-1*α* gene expression on *Tutoplast* was significantly (*P* < 0.05) reduced in comparison to reference MSC (Figure 6(c)). Osteogenic conditions lead to a further significant reduction of collagen-1*α* gene expression of MSC cultured on *Tutoplast* (*P* < 0.05, Figure 6(c)). In contrast, a trend towards increased collagen expression was observed in coculture with EPC in comparison to MSC cultured on *Tutoplast* (*P* < 0.09). Furthermore, the collagen gene expression in the coculture was significantly elevated to MSC, respectively, and MSC and EPC cultured under osteogenic conditions (*P* < 0.05, Figure 6(c)). Coculture under osteogenic conditions expressed higher collagen level in comparison to MSC under osteogenic conditions.

The gene expression of ALP (alkaline phosphatase) was significantly increased in reference MSC in comparison MSC cultured on *Tutoplast* (*P* < 0.05, Figure 6(d)).

No differences regarding the gene expression of BGLAP (osteocalcin) and RUNX (cbfa1) between the groups were observed (Figures 6(e) and 6(f)).

## 4. Discussion

In the present study we demonstrated that EPC can be cultured successfully on the processed human bone allograft *Tutoplast*, alone and in combination with MSC. 

A decline in the number of EPCs and endothelial function (DiLDL uptake) was observed over the observation period but generally the endothelial properties measured were maintained, but to a reduced level. The addition of osteogenic substances and the coculture with MSC lead to an impairment of endothelial characteristics. 

The number of adhering MSCs also declined significantly over time whereas the metabolic activity increased significantly with osteogenic stimulation. This was associated with a decrease of osteogenic differentiation (reduced collagen-1*α* gene expression) and an increase of VEGF gene expression. 

### 4.1. Clinical Use of Cancellous Bone Allografts

In a recent study the outcomes of treatment with processed cancellous bone allografts (*Tutoplast*) versus autologous bone graft obtained from the iliac crest were compared in patients with comminuted distal radius fractures. Over 70% of the patients had good to excellent outcomes in both groups [28]. This study demonstrated that processed human cancellous bone allografts can be as effective as autologous bone grafts for treating distal radius fractures [28]. In another study cranioplasty using the *Tutoplast* technology for autogenic bone processing was compared to conventional polymethylmethacrylate (PMMA) calvarial reconstruction. In general comparable results were achieved with the autogenic bone but in young patients bone resorption occurred [29].

### 4.2. Role Surface Properties Play in Cell Adhesion and Function

The favourable results with *Tutoplast* in clinical settings attributed this effect to its “natural” surface characteristics. Such a surface is thought to promote ingrowth, adherence, and migration of bone forming and other supplementary cells. Studies analyzing the influence of different biomaterials on cellular function demonstrate that cell-to-cell and cell-to-matrix interactions are key to bone formation [30, 31].

A recent *in vitro* study demonstrated that different methods of sterilizing bone grafts significantly alter MSC adhesion, proliferation, and differentiation [24]. In a former study and in the present study we confirm and extend those findings. We found that the number and metabolic activity of MSCs adhering to *Tutoplast* were superior to the synthetic materials tested [20]. Our SEM analysis revealed that *Tutoplast's* surface, consisting of a dense mesh of collagen fibrils (Figure 1(b)), is suitable for the adherence of MSC. 

Niemeyer and colleagues reported a higher seeding efficiency of MSC on collagen-1 scaffolds coated with hydroxyapatite in comparison to a *α*-TCP matrix. They also observed that MSC cultured on the collagen scaffold displayed a higher gene expression of bone sialoprotein, BMP-2, ALP, and osteocalcin when compared to MSC cultured on uncoated *α*-TCP [32]. We also found a significant improvement in adhesion and metabolic activity of MSC cultured on a collagen scaffold (*Tutoplast*) versus synthetic scaffolds such as *β*-TCP and *α*-TCP [20].

### 4.3. EPC on *Tutoplast*

In the present study we observed a significant decline in EPC adherence to *Tutoplast* over time. The relative number of adhering cells (as assessed by fluorescence microscopy) was approximately halved over the 10-day observation period. During the same time period EPC endothelial characteristics declined but persisted. 

The observed decline of the DiLDL uptake was possibly due to the cultivation medium used. In order to meet the requirements of both EPC and MSC, that is, to maintain endothelial differentiation of EPC and prevent endothelial differentiation of MSC, the medium consisted of one part of EGM + EGM2 singlequots and two parts of *MesenCult*. 

The EGM2 singlequots contains various growth and differentiating factors such as VEGF and insulin-like growth factor (IGF). Due to the addition of *MesenCult* the concentrations of both factors were reduced to one third. Both factors were necessary to maintain the state of differentiation of EPC and support the DiLDL uptake of EPC (VEGF: [33], IGF: [34]). In previous work we demonstrated that MSC cultured with this mixture did not express endothelial characteristics with the exception of VEGF gene expression [25]. 

Interestingly, we observed a significant increase of vWF gene expression in EPC* on Tutoplast*, perhaps due to the collagen surrounding. Usami et al. as well as Schmeisser et al. cultured early EPC in collagen hydrogels for at least 14 days and observed a significant vWF expression. However, no comparisons to other matrices were performed [25, 35, 36]. To our knowledge no other report is available that describes the regulation of early EPC cultivated on a dense 3D collagen-1*α* surface.

### 4.4. Effect of Osteogenic Conditions on MSC Cultured on *Tutoplast*

In the present study we observed that the number of MSC was equal under osteogenic conditions; however, their metabolic activity was significantly increased. This latter observation could be due to the transformation to an osteogenic phenotype which might be associated with a raised biosynthesis of peptides. Interestingly, the increase in metabolic activity was not accompanied by an elevated gene expression of collagen-1*α*. It was reported for dermal fibroblasts that the collagen synthesis is negatively regulated by interaction with collagen fibers via a certain collagen receptor, and mesenchymal stem cells also express collagen receptors [37, 38]. Thus, it is feasible to assume that the collagen biosynthesis underlies a similar negative feedback loop in MSC seeded onto *Tutoplast.*

### 4.5. Effect of Osteogenic Conditions on EPC Cultured on *Tutoplast*

Osteogenic substances improve the bone forming capacity of MSC but may harm the endothelial differentiation of EPC. Less information is available about the influence of osteogenic differentiation on early EPC. The current study demonstrated that the endothelial differentiation of early EPC on *Tutoplast* is partly impaired under osteogenic conditions. The DiLDL uptake was decreased under osteogenic conditions, and the gene expression of vWF tended to decline but remained still higher compared to reference EPC cultivated on a fibronectin-coated surface. Gene expression of VEGF tended to increase on *Tutoplast* under osteogenic conditions. The perpetuation of an endothelial cell-like phenotype under osteogenic conditions underlines the missing potential of early EPC for osteogenic transdifferentiation. 

In one study the effect of osteogenic stimuli on CD133 expressing late EPC was evaluated. The authors described a transformation to a osteogenic phenotype [39]. However as described in the introduction those cells differ in many respects from early EPC used in the present work (for review [40]). 

### 4.6. Differentiation in Coculture, Gene Expression Analysis

In coculture a significant decline of the vWF gene expression was observed in comparison to EPC cultured alone on *Tutoplast*. In coculture experiments a mixture consisting of each 2.5 × 10^5^ MSC and 2.5 × 10^5^ EPC was seeded; in single culture experiments 5 × 10^5^ EPCs were used. Hence, the number of EPCs was halved in comparison to single-culture experiments. This may explain the decline of vWF gene expression observed in coculture experiments. Since the vWF-gene expression of MSC is rather neglectable (10% of vWF gene expression in EPC) [25], it is feasible to assume that EPCs are the main producers of vWF mRNA in our coculture experiments.

The collagen-1*α* expression was significantly enhanced in coculture experiments. Since our own previous data demonstrated that early EPCs do not express collagen-1*α* [25], the increase of collagen-1*α* mRNA could be attributed to MSC. 

The increase of collagen-1*α* gene expression in our coculture experiments might be due to a release of osteogenic substances by EPC. A similar observation was made by Kaigler et al. They described the osteogenic stimulation of MSCs which were cocultured with endothelial cells and identified BMP2 released by the endothelial cells as a responsible factor for the osteogenic differentiation [41]. 

With regard to our study it is feasible to assume that early EPCs also release osteogenic substances (i.e., BMPs) or substances which foster the collagen synthesis (i.e., TGF-*β*) of MSC. However, if early EPCs release BMP-2 and/or TGF-*β*, this is still not known.

## 5. Conclusion

The possibility for cell-based therapy of large bone defects gains increased attention, and it has been demonstrated that the combination of MSC and EPC leads to a superior bone healing response in comparison to single-cell populations in different animal models [5, 36]. Additionally, other studies suggest an additional beneficial effect of an osteogenic differentiation of the MSC prior the implantation to the host [6–8]. However, less attention has been drawn to the carrier material to immobilize regenerative cells to the defect zone. Recent studies of our group suggest that processed cancellous bone chips are superior to synthetic bone grafts in terms of adhesion and function of MSC [20]. 

The current study extends those findings. The study provides evidence that the combined cultivation of two types of progenitor cells on a mineralized bone matrix for the purpose of tissue engineering is technically possible. The early EPCs maintain endothelial differentiation even under osteogenic conditions though endothelial activity was found to be reduced. Moreover, our results suggest that concomitant early EPCs stabilize the collagen-1*α* synthesis of MSC which might be beneficial in bone healing. However, wether MSC and EPC seeded together on a cancellous bone granules improve the bone healing has to be proved in experimental bone healing models.
